# Prevention of noise-induced hearing loss by calpain inhibitor MDL-28170 is associated with upregulation of PI3K/Akt survival signaling pathway

**DOI:** 10.3389/fncel.2023.1199656

**Published:** 2023-07-06

**Authors:** Ruosha Lai, Qiaojun Fang, Fan Wu, Song Pan, Khujista Haque, Su-Hua Sha

**Affiliations:** ^1^Department of Pathology and Laboratory Medicine, Medical University of South Carolina, Charleston, SC, United States; ^2^Department of Otolaryngology, Head and Neck Surgery, The Second Xiangya Hospital of Central South University, Changsha, Hunan, China

**Keywords:** prevention of noise-induced hearing loss, calpain inhibitor MDL-28170, PI3K/Akt signaling, alpha-fodrin, auditory sensory hair cells

## Abstract

**Introduction:**

Noise-induced calcium overload in sensory hair cells has been well documented as an early step in the pathogenesis of noise-induced hearing loss (NIHL). Alterations in cellular calcium homeostasis mediate a series of cellular events, including activation of calcium-dependent protein kinases and phosphatases. Using cell-membrane- and blood-brain-barrier-permeable calpain-1 (μ-calpain) and calpain-2 (m-calpain) inhibitor MDL-28170, we tested the involvement of calpains, a family of calcium-dependent cysteine proteases, and the potential of MDL-28170 in preventing NIHL.

**Methods:**

CBA/J mice at the age of 12 weeks were exposed to broadband noise with a frequency spectrum from 2–20 kHz for 2 h at 101 dB sound pressure level to induce permanent hearing loss as measured by auditory brainstem response and distortion product otoacoustic emissions. Morphological damage was assessed by quantification of remaining sensory hair cells and inner hair cell synapses 2 weeks after the exposure.

**Results:**

MDL-28170 treatment by intraperitoneal injection significantly attenuated noise-induced functional deficits and cochlear pathologies. MDL-28170 treatment also prevented noise-induced cleavage of alpha-fodrin, a substrate for calpain-1. Furthermore, MDL-28170 treatment prevented reduction of PI3K/Akt signaling after exposure to noise and upregulated p85α and p-Akt (S473) in outer hair cells.

**Discussion:**

These results indicate that noise-induced calpain activation negatively regulates PI3K/Akt downstream signaling, and that prevention of NIHL by treatment with MDL-28170 is associated with upregulation of PI3K/Akt survival signaling pathways.

## Introduction

Calcium (Ca^2+^) overload into sensory hair cells after traumatic noise exposure has been well documented (Maurer et al., 1993; Fridberger et al., 1998; Wu et al., 2022). Dysregulation of Ca^2+^ might lead to the activation of cysteine proteases, such as calpains, that then contribute to noise-induced hair cell death (Wang et al., 2007; Tona et al., 2014; Yamaguchi et al., 2017). Since mammalian cochlear hair cells do not regenerate, loss of hair cells causes permanent hearing loss. Currently, there are no clinically available drugs for prevention or treatment of noise-induced hearing loss (NIHL) owing to the lack of a detailed knowledge of cellular mechanisms.

Of the calpain family, calcium-dependent proteolytic enzymes, calpain-1 and calpain-2 are ubiquitously present in mammalian cells and well-studied (Spinozzi et al., 2021). These two main isoforms, calpain-1 (μ-calpain) and calpain-2 (m-calpain), require micromolar or millimolar concentrations of Ca^2+^, respectively, for activation when assessed in *in vitro* (Ono et al., 2016). Unlike other proteases, such as caspases involved in the ubiquitin-proteasome system, calpain mediated proteolysis regulates a wide range of physiological functions (Guroff, 1964; Ono et al., 2016). However, proteolysis by excessively activated calpain-1 and calpain-2 has been implicated in various pathological conditions, such as Alzheimer’s disease (Diepenbroek et al., 2014; Saito et al., 2014), neuronal injury and cell death (Lipton, 1999; Glass et al., 2002; Amini et al., 2013), and ischemia-related disorders (Yokota et al., 1999). Hence, targeting calpains has been considered a therapeutic approach in multiple settings.

Calpain inhibitors have been investigated in preclinical studies as potential therapeutic agents (Donkor, 2020). These inhibitors can be broadly separated into blood-brain barrier (BBB)-permeable calpastatin-based peptidomimetics and non-peptide inhibitors. Among them, MDL-28170 is a potent cell permeable calpain-1 and calpain-2 inhibitor that crosses the BBB. It shows neuroprotective effects against spinal cord injury, focal cerebral ischemia, and neonatal hypoxia-ischemia in rats (Northington et al., 2011).

Several calpain inhibitors have already been tested for prevention of inner ear insults. For example, leupeptin, a non-specific calpain inhibitor with limited ability to cross the BBB (Aoyagi et al., 1969), prevents gentamicin-induced cochlear and vestibular hair cell loss in cochlear explants (Ding et al., 2002). Application of MDL-28170 likewise reduces gentamicin-induced hair cell loss in explants (Lanzoni et al., 2005) and attenuates noise-induced hearing loss in guinea pigs when directly delivery to cochlear fluids (Tona et al., 2014). Treatment with a dual inhibitor of calpain and lipid peroxidation, BN82270, may attenuate NIHL (Wang et al., 2007). Additionally, the cell-permeable calpain inhibitor PD150606 ameliorates NIHL in mice treated via injection into the inner ear (Yamaguchi et al., 2017). While these results support calpain inhibitors as agents to attenuate inner ear insults, the detailed downstream reactions through which calpain might act have not been fully investigated.

While over-activation of calpains can induce cell death via caspase- or cathepsin-associated pathways (Wood and Newcomb, 1999; McCollum et al., 2002), it might also reduce phosphoinositide-3 kinase (PI3K) activity and negatively regulate the PI3K/Akt pathway (Beltran et al., 2011). In this context, an association between calpain activation and the PI3K/Akt pathway in sensory hair cells is not clear. However, our laboratory and other groups have shown that PI3K/Akt signaling pathways are involved in several forms of acquired hearing loss, including aminoglycoside-induced ototoxicity (Chung et al., 2006; Jiang et al., 2006), age-related hearing impairment (Sha et al., 2010), noise trauma (Chen et al., 2015), and other inner ear insults (Haake et al., 2009). Considering NIHL, we have previously demonstrated that the p85α regulatory subunit of the PI3K family is decreased in sensory hair cells after traumatic noise exposure and is associated with noise-induced death of outer hair cells (OHCs) (Chen et al., 2015). One of the main targets of PI3K is Akt, whose phosphorylation on serine 473 (p-Akt S473) and threonine 308 (p-Akt T308) creates a major hub in many anti-apoptotic pathways (Paez and Sellers, 2003). Indeed, p-Akt S473 decreased in sensory hair cells after inner ear insults, including aminoglycoside- and noise-induced hearing loss, as well as age-related hearing impairment (Jiang et al., 2006; Sha et al., 2010; Chen et al., 2015).

In this study, we have manipulated calpain activity using MDL-28170 treatment in order to test our hypothesis that noise-induced calcium overload activates calpain in sensory hair cells leading to loss of these cells and their function by inhibiting the PI3K/Akt signaling pathway. We first tested the potential of MDL-28170 as a protectant against of noise-induced pathologies and auditory functional deficits. We then analyzed noise-induced alterations of cleaved α-fodrin, p85α, and p-Akt (S473) in OHCs with and without MDL-28170 treatment to delineate involvement of potential pathways.

## Materials and methods

### Animals

**FIGURE 1:**
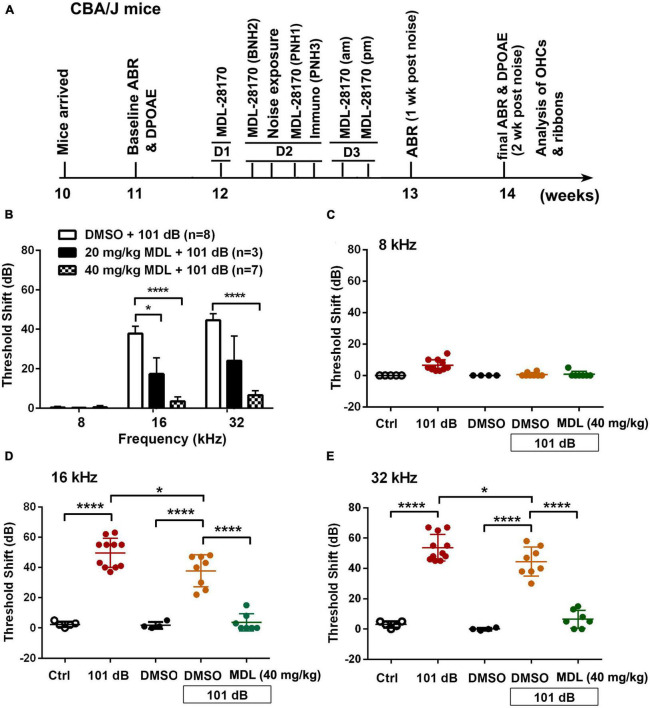
Treatment with MDL-28170 attenuates noise-induced hearing loss. **(A)** Timeline of the *in vivo* experiments: CBA/J mice arrived at MUSC at the age of 10 weeks. Baseline ABRs were measured at the age of 11 weeks. At the age of 12 weeks, 1 day before noise exposure (D1), mice received the first dose of MDL-28170. On day 2 (D2), mice received a second dose of MDL-28170 two h before noise exposure, followed by noise exposure for 2 h. Mice received a third dose of MDL-28170 one h after noise exposure. Some mice were euthanized 3 h after the exposure for immunolabeling. Mice for hair cell and ribbon counts received additional two doses (am and pm) of MDL-28170 on day 3 (D3). After final ABR and DPOAE measurements (2 weeks after the noise exposure, at the age of 14 weeks), mice were euthanized for surface preparations for hair cell counts (left ear) and ribbon counts (right ear). **(B)** MDL treatment prevents noise-induced auditory threshold shifts with the dose of 40 mg/kg to a greater extent than 20 mg/kg when measured 2 weeks after exposure. DMSO was used as the vehicle control. Data are presented as means + SD. The *n* of each animal per group is denoted in the labels. For detailed Tukey’s multiple comparisons see Table 1A. Since 20 mg/kg MDL showed minimal effects against NIHL, we did not continue to study this dose. **(C–E)** Noise exposure induced significant auditory thresholds shift at 16 and 32 kHz compared to control mice without exposure. DMSO treatment mildly attenuated the shifts at 16 kHz but not at 32 kHz. MDL treatment at 40 mg/kg furtherly attenuated the shifts at 16 and 32 kHz. Data are presented as individual points ± SD, *p < 0.05, ****p < 0.0001. For detailed Tukey’s multiple comparisons see Table 1B.

Male CBA/J mice (stock #00656) at 10 weeks of age were purchased from The Jackson Laboratory. All mice had free access to water and a regular mouse diet (Irradiated Lab Diet #5V75) and were kept at 22 ± 1°C under a standard 12:12 h light-dark cycle to acclimate for 1 week before assessing baseline auditory brainstem responses (ABRs). Hearing tests including ABRs and distortion product oto-acoustic emissions (DPOAEs) were remeasured 2 weeks after exposure to noise. Mice were exposed to noise at the age of 12 weeks and euthanized via cervical dislocation 3 h after noise exposure for assessments of molecular signaling, or at 2 weeks after noise exposure following the final ABR/DPOAE auditory functional measurements before recovery from anesthetics for hair cell morphological analysis. Figure 1A illustrates the detailed timeline of the study. In total, 165 male CBA/J mice have been used in this study. In our pilot study, we tested six groups (Ctrl, 101 dB, Ctrl + DMSO, 101 dB + DMSO, MDL 20 mg/kg + 101 dB, MDL 40 mg/kg + 101 dB). Since 20 mg/kg + 101 dB showed minimal prevention of noise-induced hearing loss, we continued the study with the five remaining groups (Ctrl, 101 dB, Ctrl + DMSO, 101 dB + DMSO, MDL 40 mg/kg + 101 dB). All specific pathogen-free mice were housed in the animal facility with controlled ambient noise levels (below 60 dB SPL) at the Medical University of South Carolina (MUSC). All research protocols were approved by the Institutional Animal Care and Use Committee at MUSC (protocol # IACUC-00752). Animal care was under the supervision of the Division of Laboratory Animal Resources at MUSC.

### Noise exposure

As in our prior experiments (Chen et al., 2012; Zheng et al., 2014; Yuan et al., 2015), we adapted our standard protocols as follows “unrestrained male CBA/J male mice at the age of 12 weeks (one mouse per stainless steel wire cage, approximately 9 cm^3^) were exposed to broadband noise (BBN) with a frequency spectrum of 2–20 kHz at 101–102 dB sound pressure level (SPL) for 2 h to induce permanent hearing loss. Noise exposures were conducted during the daytime (between 9 am–2 pm) to avoid confounding influences of circadian rhythm on hearing function. The sound exposure chamber was fitted with a loudspeaker (model 2450H; JBL) driven by a power amplifier (model XLS 202D; Crown Audio) fed from a CD player (model CD-200; Tascam TEAC American). Audio CD sound files were created and equalized with audio editing software (Audition 3; Adobe System, Inc.). The background sound intensity of the environment surrounding the cages was 65 dB as measured with a sound level meter (model 1200; Quest Technologies). Sound levels for noise exposure were measured with a sound level meter at multiple locations within the sound chamber to ensure uniformity of the sound field and measured before and after exposure to ensure stability. Control mice were kept in silence (without use of the loudspeaker) within the same chamber for 2 h.” We only used male CBA/J mice at the age of 12 weeks in these experiments to eliminate sex as confounding factor in NIHL.

### Drug administration via intra-peritoneal route to mice

MDL-28170 was purchased from Selleckchem (Cat# S7394) and dissolved in dimethyl sulfoxide (DMSO) as a stock solution (40 mg/mL) and stored at −20°C. The stock solution was diluted with 0.9% saline solution immediately before injections. In our pilot experiments, we tested two doses of MDL-28170 (20 mg/kg and 40 mg/kg) for prevention of NIHL based on prior literature (Thompson et al., 2010). Since 20 mg/kg MDL-28170 showed minimal effects against noise-induced hearing loss, we did not continue to study this dose. We used MDL-28170 at 40 mg/kg for the experiments. Vehicle control mice received the same volume of DMSO in saline. For assessments of signals by immunohistochemistry and Western blots, each animal received a total of three intraperitoneal (IP) injections of MDL-28170 at a dose of 40 mg/kg per injection. Three IP injections were administered 1 day before, 2 h before, and 1 h after noise exposure. The mice used for experiments to observe the effects of treatment on ABR thresholds and DPOAE amplitudes received two additional IP injections on the day following noise exposure (am and pm).

### Auditory brainstem response and distortion product otoacoustic emission measurements

Auditory brainstem responses (ABRs) and DPOAEs were performed in the left ears of anesthetized mice 1 week before and 2 weeks after noise exposure. As in our prior experiments (Chen et al., 2012; Zheng et al., 2014; Yuan et al., 2015), we adapted our standard protocols as follows “mice were anesthetized with an intraperitoneal (IP) injection of a mixture of ketamine (100 mg/kg) and xylazine (10 mg/kg), and then placed in a sound-proof and electrically-shielded booth (Acoustic Systems). Body temperature was monitored and maintained near 37°C with a heating pad. Acoustic stimuli were delivered monaurally to a Beyer earphone attached to a customized plastic speculum inserted into the ear canal. Subdermal electrodes were inserted at the vertex of the skull (active), mastoid region under the left ear, and mastoid region under the right ear (ground). ABRs were measured at 8, 16, and 32 kHz. Tucker Davis Technology (TDT) System III hardware and SigGen/Biosig software were used to present the stimuli (15 ms duration tone bursts with 1 ms rise-fall time) and record the response. Up to 1,024 responses were averaged for each stimulus level. Thresholds were determined for each frequency by reducing the intensity in 10-dB increments and then in 5-dB steps near threshold until no organized waves were detected. Thresholds were estimated between the lowest stimulus level where a wave was observed and the highest level without wave. ABR wave II was used to determine thresholds for each frequency and thresholds were assigned by an expert who was blinded to the treatment conditions. DPOAE testing was performed following ABR measurements. The emissions were measured using TDT RZ6 System and SigGen software. The acoustic assembly containing an ER-10B+ microphone connected to two transducers was tightly apposed into the ear canal. Primary tones were presented at fixed intensity levels of L1 = 65 dB SPL and L2 = 55 dB SPL with f2/f1 ratio of 1.2. Physiological responses of each mouse were analyzed for individual frequencies and averaged for each of these frequencies from 4 kHz to 40 kHz. After ABR and DPOAE measurements, mice were placed in individual cages with warmed blue pads for beds until completely recovered from anesthesia. After final auditory functional assessments, mice were euthanized for surface preparations. Left ears were used for hair cell counts and right ears for ribbon counts.”

### Immunohistochemistry for cochlear surface preparations

We have followed a procedure as previously described and used both ears for immunohistochemistry (Chen et al., 2012; Zheng et al., 2014; Yuan et al., 2015). Our standard protocols as follows “the temporal bones were removed and perfused locally with a solution of 4% paraformaldehyde in phosphate-buffered saline (PBS), pH 7.4, and kept in this fixative overnight at 4°C. Between each step, the cochlear samples were washed at least three times with PBS for 5–10 min each wash. After decalcification with 4% sodium EDTA solution (adjusted with HCl to pH 7.4) for 3 days at 4°C, the cochleae were micro-dissected into three turns (apex, middle, and base) and adhered to 10-mm round coverslips (Microscopy Products for Science and Industry, #260367) with Cell-Tak (BD Biosciences, Franklin Lakes, NJ, USA, #354240). The specimens were first permeabilized in 2% Triton X-100 solution and then blocked with 10% normal goat serum for 30 min each step at room temperature, followed by incubation with primary antibodies: polyclonal rabbit anti-Calpain I (Abcam, Cambridge, UK, #ab39170, 1:50), polyclonal rabbit anti-Calpain II (Abcam #ab39165, 1:50), cleaved α-fodrin (Cell Signaling Technology, Danvers, MA, USA, #2121, 1:50), monoclonal rabbit anti-PI3K, p85α (Millipore, Burlington, MA, USA, #04-403, 1:50), monoclonal rabbit anti-p-Akt (Ser473) (Cell Signaling Technology #4060, 1:50) at 4°C for overnights (24–48 h). The specimens were then incubated with the Alexa-Fluor-594-conjugated secondary antibody at a concentration of 1:200 overnight at 4°C and followed by incubation with Alexa Fluor™ 488 Phalloidin (Thermo Fisher, Waltham, MA, USA, #A12379, 1:200) overnight at 4°C in darkness. Control incubations were routinely processed without primary antibody treatments.

Ribeye is a component of synaptic ribbons. CtBP2 and ribeye share an epitope that is recognized by the anti-CtBP2 antibody. Surface preparations for assessments of inner hair cell (IHC) ribbon synapses, the specimens were incubated in darkness at 37°C overnight with primary monoclonal mouse anti-CtBP2 IgG1 at 1:200 (BD Biosciences, #612044) and mouse anti-GluA2 IgG2a at 1:2,000 (Millipore, #MAB397), followed by the Alexa-Fluor-594 goat anti-mouse IgG1 and Alexa-Fluor-488 goat anti-mouse IgG2a (1:1,000) at 37°C for 1 h in darkness (Wan et al., 2014; Hill et al., 2016).

Surface preparations for counting hair cells were incubated with myosin-VIIa (Proteus Biosciences, Ramona, CA, USA, #25-6790, 1:200) overnight at 4°C and then incubated overnight at 4°C with secondary antibody (biotinylated goat anti-rabbit) at a 1:100 dilution. The specimens were then incubated in ABC solution (Vector Laboratories, Newark, CA, USA, #PK-4110) overnight followed by incubation in 3,3′-diaminobenzidine (DAB) for 3 h, as necessary for sufficient staining intensity. Finally, the specimens were washed to stop the DAB reaction.

After at least three final washes with PBS, all immunolabeling samples (already on round coverslips) were mounted by adding 8 μL mounting agent (Fluoro-gel with Tris buffer, Electron Microscopy Sciences, Hatfield, PA, USA, #17985-10), sandwiched with another round coverslip, and placed on a microscope slide (Fang et al., 2019). Finally, edges were sealed with nail polish. Immunolabeled images were taken with a 63 × -magnification lens under identical Z-stack conditions using Zeiss LSM 880.”

### Semi-quantification of the immunolabeling signals from outer hair cells of surface preparations

Immunohistochemistry captured by confocal images for semi-quantitative analysis is well accepted methodology when used with careful consideration of the utility and semi-quantitative nature of the assays (Taylor and Levenson, 2006; Walker, 2006). As in our prior experiments (Chen et al., 2012; Zheng et al., 2014; Yuan et al., 2015), we adapted our standard protocols as follows “Immunolabeling for cleaved α-fodrin, p-p85α, and p-Akt (S473) was semi-quantified from original confocal images with 8-bit grayscale values, each taken with a 63 × -magnification lens under identical conditions and equal parameter settings for laser gains and photomultiplier tube (PMT) gains within linear ranges of the fluorescence, using Image J software (National Institutes of Health, Bethesda, MD, USA). The cochleae from the different groups were fixed and immunolabeled simultaneously with identical solutions and processed in parallel. All surface preparations were counterstained with phalloidin (green) to identify the comparable parts of the OHC in confocal images. The regions of interest of individual OHCs were outlined with the circle tool based on phalloidin staining. The immunolabeling in grayscale in OHCs was measured in the upper-basal region of surface preparations (corresponding to sensitivity to 22–32 kHz) in 0.12-mm segments, each containing about 60 OHCs. The intensity of the background was subtracted and the average grayscale intensity per cell was then calculated. For each repetition, the relative grayscale value was determined by normalizing the ratio to control. Since there were no significant changes in all assessed immunolabeling in the apex and middle regions of cochlear OHCs when assessed 3 h after the completion of noise exposure, we performed only semi-quantification of the immunolabeling signals from OHC in the basal turn (corresponding to sensitivity to 22–32 kHz) by a person who was blind to the treatment groups.” This procedure provided semi-quantitative measurements that are not confounded by protein expression in other cell types of the cochlea. To be clear, the confocal images used to compare fluorescence intensity must be acquired under the same settings and the same processing enhancement for the captured images is used. To prevent bias, the person analyzing images must be blind to the treatment groups.

### Semi-quantification of the immunolabeled ribbons from Z projections on surface preparations

We have followed a procedure as previously described (Hill et al., 2016). Since we have not observed obviously “orphaned” GluA2-labeled post-synaptic terminals 14 d after exposure to noise, we only counted CtBP2 labeled pre-synaptic ribbons (red dots). Our standard protocol is as follows “Immunofluorescence of CtBP2 on surface preparations was quantified from original confocal images, each taken with a 63 × -magnification lens under identical Z-stack conditions in 0.25-mm intervals and equal parameter settings for laser gains and PMT gains. The z-stack images in each 0.12-mm segment (containing about 16 IHCs) were captured from cochlear surface preparations. The number of synaptic ribbons were counted using ImageJ software (National Institutes of Health, Bethesda, MD, USA). Briefly, the background of the images was subtracted, the noise was despeckled once, and the threshold was set to isolate the immunolabeling of ribbon signals. The image was then converted to a binary file and the number of ribbon particles was counted using the 3D Object Counter and divided by the total number of IHC nuclei within the image.”

### Counts of hair cells

As in our prior experiments (Chen et al., 2012; Zheng et al., 2014; Yuan et al., 2015), we adapted our standard protocols as follows “Images from the apex through the base of the myosin-VIIa-labeled and DAB-stained surface preparations were captured using a 20 × lens on the Zeiss microscope. The lengths of the cochlear epithelia were measured and recorded in millimeters. Mapping of frequencies as a function of distance along the entire length of the cochlear spiral was calculated with the equation [d (%) = 156.5–82.5 × log (f)] from Müller’s paper (Muller et al., 2005). The results are in agreement with the literature (Viberg and Canlon, 2004). OHCs were counted from the apex to the base along the entire length of the mouse cochlear epithelium. The percentage of hair cell loss in each 0.5-mm length of epithelium was plotted as a function of the cochlear length as a cytocochleogram.”

### Extraction of total cochlear protein from mouse cochleae

As in our prior experiments (Chen et al., 2012; Zheng et al., 2014; Yuan et al., 2015), we adapted our standard protocols as follows “Both inner ears were rapidly removed and dissected in ice-cold PBS, pH7.4, containing complete™ mini EDTA-free protease inhibitor cocktail tablets (Sigma-Aldrich, Burlington, MA, USA, #11836170001) to remove vestibular portions. To extract total protein, tissues from two cochleae of a mouse were homogenized in ice-cold radioimmunoprecipitation assay lysis buffer (Sigma-Aldrich, R0278) with the cocktail proteinase inhibitors plus Phosphatase Inhibitor Cocktails II and III (Sigma-Aldrich, #P5726 and #P0044) by using a glass/glass micro–tissue grind pestle and vessel for 30 s. After 30 min on ice, tissue debris was removed by centrifugation at 15,000 × *g* at 4°C for 10 min and the supernatants were retained as the total protein fractions. Protein concentrations were determined using the Bio-Rad Protein Assay dye reagent (Bio-Rad, Hercules, CA, USA, #500-0114) with bovine serum albumin as a protein standard. Finally, the total protein was stored at −80°C after quantification.”

### Western blot analysis

As in our prior experiments (Chen et al., 2012; Zheng et al., 2014; Yuan et al., 2015), we adapted our standard protocols as follows “Protein samples (30 μg) were separated by SDS-polyacrylamide gel electrophoresis. After electrophoresis, the proteins were transferred onto a nitrocellulose membrane (Pierce) and blocked with 5% solution of non-fat dry milk in PBS-0.1% Tween 20 (PBS-T). The membranes were incubated with anti-α-fodrin (Cell Signaling Technology #2122, 1:1,000), anti-p-Akt (S473) (Cell Signaling Technology #4060 1:1,000), anti-total Akt1/2 (Cell Signaling Technology #9272, 1:1,000), or anti-glyceraldehyde 3-phosphate dehydrogenase (GAPDH) (Millipore #ABS16, 1:10,000) at 4°C overnight, and then washed three times (10 min each) with PBS-T buffer. Membranes were then incubated with the appropriate secondary antibody at a concentration of 1:2,500 for 1 h at room temperature. Following extensive washing of the membrane, the immunoreactive bands were visualized by SuperSignal^®^ West Dura Extended Duration Substrate or Pierce^®^ ECL Western Blotting Substrate (Thermo Scientific).

Western blot bands were scanned by LI-COR Odyssey Fc imaging system and analyzed using Image J software. First, the background staining density for each band was subtracted from the band density. Next, the probing protein/GAPDH ratio was calculated from the band densities run on the same gel to normalize for differences in protein loading. Finally, the difference in the ratio of the control and experimental groups was tested for statistical significance.”

### Statistical analyses

As in our prior experiments (Chen et al., 2012; Zheng et al., 2014; Yuan et al., 2015), our standard protocol for statistical analysis is as follows “Data were analyzed using SYSTAT and GraphPad software for Windows. Biological sample sizes were determined based on the variability of measurements and the magnitude of the differences between groups, as well as experience from our previous studies, with stringent assessments of difference. Data of OHC loss along the length of the cochlear spiral were analyzed with repeated measures one-way analysis of variance (ANOVA) with *post-hoc* comparisons using SYSTAT. The rest of the analyses were done using GraphPad. Differences with multiple comparisons were evaluated by one-way ANOVA with multiple comparisons. Differences for single-pair comparisons were analyzed using two-tailed unpaired Student’s *t*-tests. Data for relative ratios of single-pair comparisons were analyzed with one-sample *t*-tests. A *p*-value < 0.05 was considered statistically significant. Data are presented as means ± SD or SEM based on the sample size and variability within groups. Sample sizes are indicated for each figure.”

## Results

### MDL-28170 treatment prevents noise-induced auditory functional deficits and sensory outer hair cell loss

**FIGURE 2:**
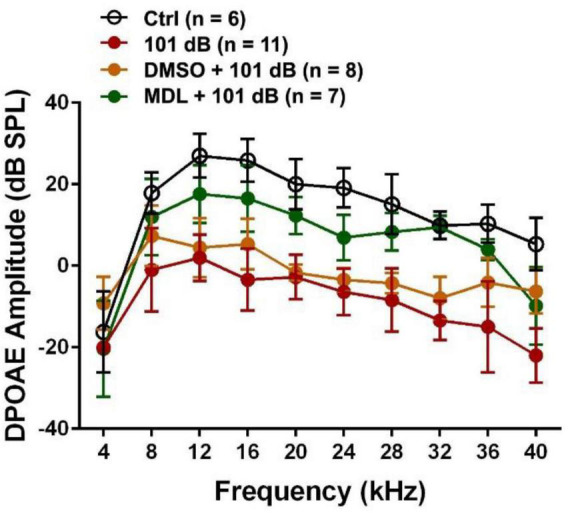
Treatment with MDL-28170 prevents outer hair cell functional deficits. DPOAE amplitudes from 8-40 kHz were significantly diminished by noise exposure, whereas MDL-28170 (40 mg/kg) treatment significantly prevented noise-induced decreases in amplitudes from 12–36 kHz. Treatment with DMSO did not prevent noise-decreased amplitudes. Data are presented as means ± SD. Detailed statistical analysis is shown in Table 2.

MDL-28170 has been used in doses of 10 to 50 mg/kg in *in-vivo* experiments (Li et al., 1998; Ai et al., 2007; Kharatmal et al., 2015). In our pilot experiments, we tested two doses, of 20 mg/kg and 40 mg/kg, with a total of five injections on three consecutive days. Mice appeared healthy with normal body weights and shiny fur comparable to the saline-treated control mice. There was also no hearing loss. We then assessed the efficacy of these two doses for attenuation of 101-dB-noise-induced auditory threshold shifts in six groups (Ctrl, 101 dB, DMSO, 101 dB + DMSO, MDL 20 mg/kg + 101 dB, and MDL 40 mg/kg + 101 dB). The dose of 20 mg/kg showed minimal prevention of NIHL, whereas the 40-mg/kg offered stronger protection at 16 (*F*_2_, _15_ = 23.79, *p* < 0.0001) and 32 kHz (*F*_2_, _15_ = 22.64, *p* < 0.0001) (Figure 1B, detailed Tukey’s multiple comparison of ABR data see Table 1A), we continued the study with the five remaining groups (Ctrl, 101 dB, DMSO, 101 dB + DMSO, and MDL 40 mg/kg + 101 dB) in all subsequent experiments used MDL-28170 at 40 mg/kg. Control mice (saline control, DMSO vehicle control, and MDL-28170 alone) without noise exposure had no hearing loss. Consistent with our previous publications (Zheng et al., 2014; Yuan et al., 2015; Hill et al., 2016; He et al., 2021; Wu et al., 2022), noise exposure for 2 h induced auditory threshold shifts averaging 7 dB at 8 kHz, 50 dB at 16 kHz, and 54 dB at 32 kHz. Differences in threshold shifts compared to controls were significant at 16 (*F*_4_, _30_ = 61.95, *p* < 0.0001), and 32 kHz (*F*_4_, _30_ = 86.28, *p* < 0.0001) but not at 8 kHz as analyzed by one-way ANOVA. The vehicle DMSO mildly attenuated threshold shifts at 16 kHz (*p* < 0.05), and no significant attenuation at 32 kHz (*p* > 0.05). Treatment with MDL-28170 nearly completely prevented the shifts at 16 (*p* < 0.0001), and 32 kHz (*p* < 0.0001) with residual average shifts of only 4 dB and 7 dB at 16 and 32 kHz, respectively (Figures 1C–E, detailed Turkey’s multiple comparisons of ABR values see Table 1B). Furthermore, noise exposure significantly diminished DPOAE amplitudes from 8 to 40 kHz (*F*_1_, _9_ = 67.970, *p* < 0.0001) and this reduction was significantly alleviated with MDL-28170 treatment (Figure 2, *F*_1_, _9_ = 9.847, *p* = 0.012; for detailed *post-hoc* statistical values of DPOAE see Table 2). Treatment with DMSO did not prevent noise-diminished DPOAE amplitudes (Figure 2).

**TABLE 1:** Tukey’s multiple comparisons of threshold shifts (Figure 1).

Frequency (kHz)	Groups	*p* value	Marker
**A: Tukey’s multiple comparison of Figure 1B ABR data.**			
16	DMSO + 101 dB vs. 20 mg/kg MDL + 101 dB	*p* < 0.05	*
	DMSO + 101 dB vs. 40 mg/kg MDL + 101 dB	*p* < 0.0001	****
32	DMSO + 101 dB vs. 20 mg/kg MDL + 101 dB	p < 0.05	ns
	DMSO + 101 dB vs. 40 mg/kg MDL + 101 dB	*p* < 0.0001	****
**B: Tukey’s multipe comparison of ABR data for Figures 1C–E**			
16	Ctrl vs. 101 dB	*p* < 0.0001	****
	DMSO vs. DMSO + 101 dB	*p* < 0.0001	****
	101 dB vs. DMSO + 101 dB	*p* < 0.05	*
	DMSO + 101 dB vs. MDL + 101 dB	*p* < 0.0001	****
32	Ctrl vs. 101 dB	*p* < 0.0001	****
	DMSO vs. DMSO + 101 dB	*p* < 0.0001	****
	101 dB vs. DMSO + 101 dB	*p* > 0.05	ns
	DMSO + 101 dB vs. MDL + 101 dB	*p* < 0.0001	****

*p < 0.05, ****p < 0.0001.

**TABLE 2:** *Post-hoc* analysis of DPOAE (Figure 2).

Groups	Frequency (kHz)	*p* value	Marker
DMSO + 101 dB vs. MDL + 100 dB	4	*p* = 0.027	ns
	8	*p* = 0.39	ns
	12	*p* = 0.014	*
	16	*p* = 0.034	*
	20	*p* < 0.0001	****
	24	*p* = 0.005	**
	28	*p* < 0.0001	****
	32	*p* < 0.0001	****
	36	*p* < 0.015	*
	40	*p* = 0.49	ns
Ctrl vs. 100 dB	4	*p* = 0.13	ns
	8	*p* = 0.0014	**
	12	*p* < 0.0001	****
	16	*p* < 0.0001	****
	20	*p* < 0.0001	****
	24	*p* < 0.0001	****
	28	*p* = 0.001	**
	32	*p* < 0.0001	****
	36	*p* < 0.0001	****
	40	*p* < 0.0001	****

*p < 0.05, ****p < 0.0001.

**FIGURE 3:**
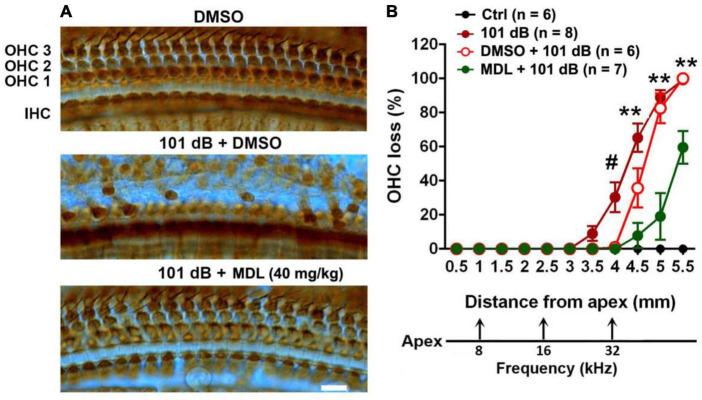
MDL treatment attenuates noise-induced outer hair cell loss. **(A)** Representative images of DAB staining in myosin-VIIa-labeled OHCs in the basal turn examined 2 weeks after noise exposure. OHC1, 2, and 3 and IHC indicate the three rows of outer hair cells and one row of inner hair cells. Scale bar = 10 μm. **(B)** Counts of noise-induced loss of OHCs along the cochlear spiral confirms a significant reduction by MDL-28170 treatment. Distances along the cochlear duct correlate with the frequencies as indicated. Data are presented as means + SD. The *n* of each group is denoted in the labels; symbol ^#^indicates 101 dB vs. DMSO + 101 dB, ^#^*p* < 0.05, symbol *indicates DMSO + 101 dB vs. MDL + 101 dB; **p < 0.01. The three groups (101 dB, DMSO + 101 dB, and MDL + 101 dB) were analyzed by one-way ANOVA with Tukey’s multiple comparisons (Table 3).

To assess the protective effect of MDL-28170 against noise-induced hearing loss at the morphological levels, we counted OHC numbers along the entire length of the cochlear spiral after final ABR measurements (2 weeks after noise exposure) and labeling with myosin-VIIa and staining with DAB. Like our previous results (He et al., 2021; Wu et al., 2022), noise-induced OHC loss followed a base-to-apex gradient with a complete OHC loss in the base of the cochlear epithelium (Figure 3). We analyzed OHC loss in six different treatment groups (saline or DMSO or MDL-28170 control without exposure, 101 dB, DMSO + 101 dB, and MDL + 101 dB). Without noise exposure, all control mice showed intact OHCs. Treatment with DMSO reduced noise-induced OHC loss with a significant reduction only at 4 mm from the apex (*p* < 0.001), while MDL-28170 treatment significantly further prevented OHC loss. At the 4.5-mm region, OHC loss was reduced from 65 to 5% (*p* < 0.001), at 5 mm from 88 to 20% (*p* < 0.001), and at 5.5 mm from 100 to 60% (*p* < 0.001) (Figure 3, for detailed one-way ANOVA and Tukey’s multiple comparison values see Table 3). While the mild attenuation by DMSO can be ascribed to its antioxidant properties, the sum of the functional and morphological results clearly indicate that MDL-28170 treatment can more effectively prevent NIHL.

**TABLE 3:** One-way ANOVA and multiple comparisons of hair cell loss (Figure 3).

Distance (mm)	F-values	Groups	*p* value	Marker
4	*F*_2,18_ = 11.65, *p* = 0.0006	101 dB vs. DMSO + 101 dB	*p* < 0.01	**
		DMSO + 101 dB vs. MDL + 101 dB	p > 0.05	ns
4.5	*F*_2,18_ = 14.55, *p* = 0.0002	101 dB vs. DMSO + 101 dB	*p* > 0.05	ns
		DMSO + 101 dB vs. MDL + 101 dB	*p* < 0.01	**
5	*F*_2,18_ = 17.12, *p* < 0.0001	101 dB vs. DMSO + 101 dB	*p >* 0.05	ns
		DMSO + 101 dB vs. MDL + 101 dB	*p* < 0.01	**
5.5	*F*_2,18_ = 17.23, *p* < 0.0001	101 dB vs. DMSO + 101 dB	*p >* 0.05	ns
		DMSO + 101 dB vs. MDL + 101 dB	*p* < 0.01	**

**p < 0.01.

### MDL-28170 treatment prevents noise-induced loss of inner hair cell synapses

**FIGURE 4:**
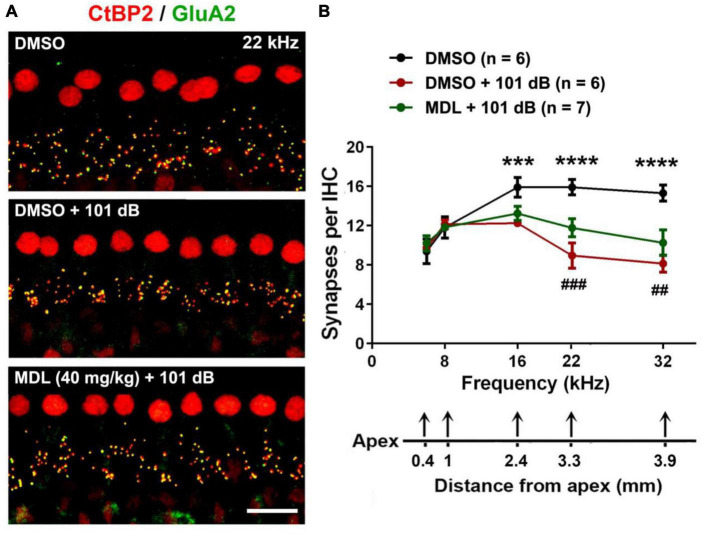
MDL-28170 treatment attenuates noise-induced loss of inner hair cell synapses. **(A)** Representative images revealed immunolabeling for CtBP2 (red) and GluA2 (green) examined 2 weeks after noise exposure. Images are comprised of 25 Z-stack projections taken in the upper basal turn corresponding to 22 kHz, scale bar = 10 μm. **(B)** Counts of CtBP2-GluA2-immunolabeled ribbon particles in IHCs corresponding to 6, 8, 16, 22, and 32 kHz revealed significant reduction at 16, 22, and 32 kHz after noise exposure, while MDL treatment attenuated the reduction of intact synapses at 22 and 32 kHz. Distances along the cochlear duct correlate with the frequencies as indicated; the *n* of each group is denoted in the labels; symbol *indicates DMSO vs. DMSO + 101 dB, symbol ^#^indicates DMSO + 101 dB vs. MDL + 101 dB; ^##^*p* < 0.01, ^###^*p* < 0.001, ***p < 0.001, ****p < 0.0001.

Noise-induced loss of IHC synapses has been well-documented in our previous publications and others (Wan et al., 2014; Hill et al., 2016; Fang et al., 2019). To test whether MDL-28170 treatment can protect against noise-induced loss of IHC synapses, we employed co-immunolabeling for CtBP2 for the presynaptic ribbon and GluA2 for the postsynaptic terminal 2 weeks after the final ABR measurements. Similar to our previous reports (Hill et al., 2016; Wu et al., 2022), exposure to 101-dB broadband noise resulted in loss of IHC synapses at 16, 22, and 32 kHz compared to control mice receiving DMSO or MDL-28170 or saline without noise exposure. All control mice showed similar numbers of IHC synapses. We then focused on three groups (DMSO only, DMSO + 101 dB, and MDL + 101 dB). One-way ANOVA analysis (Figures 4A, B) of the three groups showed significant differences in number of synapses per IHC in the regions of 0.4, 1.0, 2.4, 3.3, and 3.9 mm from the apex (corresponding to 6, 8, 16, 22, and 32 kHz) at 16 (*F*_2_, _15_ = 37.29, *p* < 0.0001), 22 (*F*_2_, _15_ = 69.08, *p* < 0.0001), and 32 kHz (*F*_2_, _15_ = 70.20, *p* < 0.0001) but not at 6 and 8 kHz. Tukey’s multiple comparison tests indicated that noise significantly reduced IHC synapses at 16 (*p* < 0.001), 22 (*p* < 0.0001), and 32 kHz (*p* < 0.0001), whereas treatment with MDL-28170 moderately attenuated noise-induced IHC synapse loss in both the 22-kHz (*p* < 0.001) and 32-kHz regions (*p* < 0.01).

### MDL-28170 treatment inhibits noise-induced cleavage of α-fodrin in cochlear tissues including outer hair cells

**FIGURE 5:**
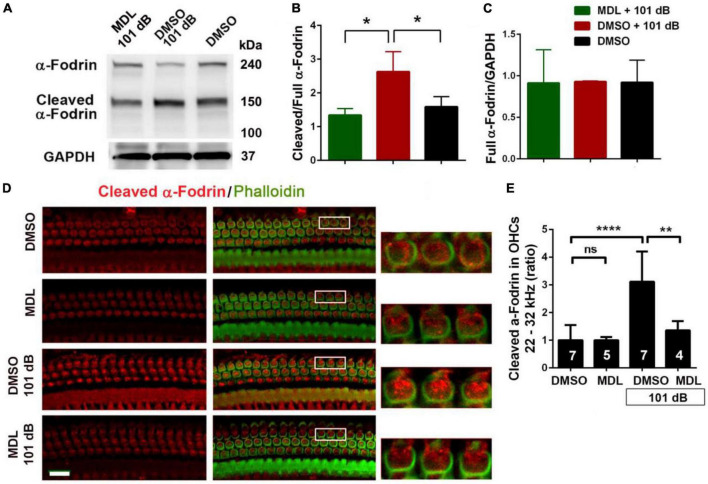
MDL-28170 treatment inhibits noise-induced α-fodrin activation in the cochlear tissues. **(A)** Representative immunoblots from whole cochlear homogenates revealed 240 kDa α-fodrin and the cleaved 150 kDa fragment 3 h after noise exposure. GAPDH (37 kDa) was used as the sample loading control. **(B,C)** Semi-quantifications of each band density show that the ratio of cleaved to full α-fodrin increased with noise exposure and MDL treatment prevented such an increase **(B)**, while the cleaved band density divided by GAPDH was not different among the three groups **(C)**. Data are presented as means + SD; *n* = 3 in each group with one mouse (two cochleae) per sample, *p < 0.05. **(D)** Representative images were taken from the basal turn of cochlear epithelia at the time point 3 h after noise exposure. MDL treatment alone showed similar immunolabeling intensity for cleaved α-fodrin (red) in OHCs as the DMSO-treated group. Noise exposure significantly increased cleaved α-fodrin in OHCs, whereas MDL treatment prevented such effects. Phalloidin (green) was a counterstain for visualization of OHCs. The enlarged OHCs allow for better visualization of the immunolabeling. Scale bar = 10 μm. **(E)** Confocal images used to compare fluorescence intensity by semi-quantification of immunolabeling for cleaved α-fodrin in OHCs were acquired under the same settings and the same processing enhancement for the captured images was used. The person analyzing images was blind to the treatment groups. It confirmed a significant increase after noise exposure, whereas MDL treatment prevented such effects. Data are presented as means + SD; the *n* is indicated in the bar for each condition with use of one cochlea per mouse. **p < 0.01, ****p < 0.0001.

In our pilot experiments, we did not observe any differences in total calpain I and II expression in OHCs at 1, 3, and 24 h after completion of noise exposure. Fodrin, a membrane-associated cytoskeletal protein, is a substrate for calpain I (Siman et al., 1984) and α-fodrin is one of the primary targets cleaved by caspases during apoptosis (Bennett and Gilligan, 1993). Noise-induced hair cell death through caspase-dependent cell death pathways has been well-documented by our and other labs (Zheng et al., 2014; Yamaguchi et al., 2017) and therefore we assessed expression of α-fodrin at 3 h after noise exposure. The ratio of cleaved (150 kDa) to full length (240 kDa) α-fodrin increased significantly after noise exposure (*p* < 0.05). This activation of α-fodrin was completely abolished by MDL-28170 treatment (Figures 5A–C). We then immunolabeled cleaved α-fodrin on cochlear surface preparations in four groups (DMSO, MDL, DMSO + 101 dB, and MDL + 101 dB) at 3 h after exposure to assess if cleaved α-fodrin localized in OHCs. Immunolabeling for cleaved α-fodrin increased in OHCs in the noise-exposed group compared to vehicle control without noise exposure. MDL-28170 treatment blocked such an increase (Figures 5D, E). Semi-quantified immunolabeling in grayscales confirmed significant differences between the four groups (*F*_3_, _19_ = 13.75, *p* < 0.0001). Noise exposure resulted in a twofold increase in cleaved α-fodrin in OHCs compared to vehicle controls without exposure (*p* < 0.0001), whereas MDL-28170 treatment inhibited this increase (*p* < 0.01). There was no difference between DMSO and MDL-28170 treatment alone without noise exposure. These results suggest that noise exposure activates calpain in the inner ear, including in sensory hair cells. In contrast, MDL-28170 treatment inhibits this activation.

### MDL-28170 treatment prevents noise-diminished PI3-K/Akt pathway signaling in outer hair cells

**FIGURE 6:**
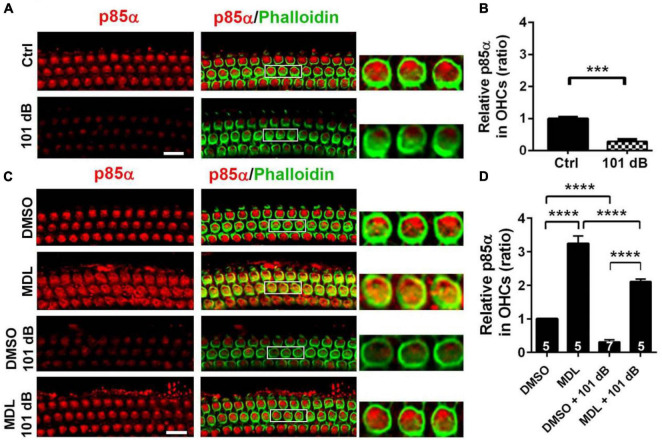
MDL-28170 treatment prevents noise-reduced p85α in outer hair cells. **(A)** Surface preparations show weak immunolabeling for p85α in OHCs in the basal turn 3 h after 101-dB noise exposure compared to controls. Phalloidin (green) was a counterstain for OHCs. The enlarged OHCs allow for better visualization of the immunolabeling. Scale bar = 10 μm. **(B)** Noise-reduced p85α immunolabeling in OHCs was confirmed by semi-quantitative analysis of grayscales of the immunolabeling. Data are presented as means ± SD, *n* = 5 in each group. **(C)** Treatment with MDL prevented noise-reduced p85α in OHCs compared to vehicle controls. MDL alone also increased p85α in OHCs without noise exposure compared to DMSO. Scale bar = 10 μm. **(D)** Semi-quantification of immunolabeling for p85α in OHCs confirmed a significant increase by MDL treatment with or without noise exposure. Data are presented as means ± SD; *n* is indicated in the bar for each condition with use of one cochlea per mouse, ***p < 0.001, ****p < 0.0001. All confocal images used to compare fluorescence intensity were acquired under the same settings and the same processing enhancement for the captured images was used. The person analyzing images was blind to the treatment groups.

**FIGURE 7:**
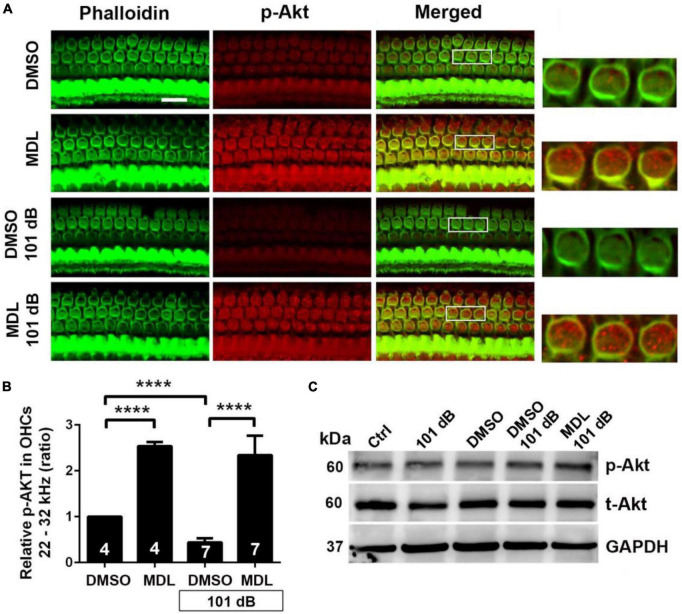
MDL treatment prevents noise-decreased p-Akt (S473) in outer hair cells. **(A)** Representative images revealed that MDL alone significantly increased p-Akt immunolabeling (red) in OHCs compared to vehicle control (DMSO group) without noise exposure when examined 3 h after noise exposure. MDL treatment prevented noise-decreased immunolabeling for p-Akt in OHCs. Phalloidin (green) was a counterstain for OHCs. The enlarged OHCs allow for better visualization of the immunolabeling. Scale bar = 10 μm. **(B)** MDL treatment alone significantly increased immunolabeling for p-Akt in OHCs per semi-quantitative the gray-scale intensity. MDL treatment prevented noise-decreased p-Akt. Data are presented as means + SD. The *n* is indicated in the bar for each condition using one cochlea per mouse, ****p < 0.0001. All confocal images used to compare fluorescence intensity were acquired under the same settings and the same processing enhancement for the captured images was used. The person analyzing images was blind to the treatment groups. **(C)** Immunoblots using whole cochlear homogenates showed a single band of p-Akt and total Akt at 60 kDa with no changes in the levels between groups. GAPDH (37 kDa) was used as the sample loading control, *n* = 4 in each group with two cochleae per sample per mouse.

Calpain activation reduces PI3-K activity and its inhibition promotes cell survival during serum starvation in cell culture (Beltran et al., 2011). Our previous studies showed that noise exposure decreased PI3-K subunits p85α, but not p110 in OHCs (Chen et al., 2015). In the meantime, p-Akt (S473) but not p-Akt (T308) also decreased in OHCs after exposure (Chen et al., 2015). To determine if MDL-28170 treatment prevents noise-induced hair cell death through PI3-K signaling, we first assessed p85α immunolabeling in OHCs. Consistent with our previous report, noise exposure decreased p85α by about 50% in OHCs 3 h after the completion of exposure (*t*_6_ = 6.1482, *p* = 0.0008, Figures 6A, B). We then immunolabeled p85α in four groups (DMSO, MDL, DMSO + 101 dB, and MDL + 101 dB) 3 h after noise exposure. MDL-28170 treatment alone without exposure significantly increased p85α twofold in OHCs compared to a DMSO vehicle control group (*t*_4_ = 24.5967, *p* < 0.0001). Moreover, MDL-28170 treatment completely prevented the decrease in p85α in OHCs by noise exposure, and significantly elevated p85α levels to double that of non-exposed naïve mice (*p* < 0.0001), although these p85α levels were still significantly lower than those achieved by MDL-28170 without noise exposure (*p* < 0.0001, Figures 6C, D). Next, we evaluated immunolabeling for p-Akt (S473) in OHCs 3 h after noise exposure. Like our previous data, immunolabeling for p-Akt (S473) decreased by 60% compared to controls without exposure (*t*_6_ = 14.3414, *p* < 0.0001). Treatment with MDL-28170 alone without noise exposure resulted in a 1.5-fold increase in p-Akt (S473) compared to DMSO alone (*t*_3_ = 34.2222, *p* < 0.0001). Importantly, MDL-28170 treatment completely prevented reduction of p-Akt (S473) (*p* < 0.0001) by noise exposure, and elevated p-Akt (S473) to levels close to treatment with MDL-28170 alone. There was no difference between p-Akt (S473) levels in MDL-28170 alone and MDL + noise exposure groups (Figures 7A, B). Immunoblots using whole cochlear homogenates 3 h after completion of exposure showed a single band for p-Akt and total Akt at 60 kDa with no changes in the levels between the five groups (Ctrl, 101 dB, DMSO, DMSO + 101 dB, and MDL + 101 dB) (Figure 7C). This may be due to the dilution by other cell types in the homogenates. These results suggest that attenuation of noise-induced hearing loss and hair cell loss by treatment with MDL-28170 is associated with upregulation of PI-3K/Akt cell survival pathways.

## Discussion

Two salient points emerge from our results. First, targeting noise-induced calpain activation is a viable approach for preventing losses of IHC ribbon synapses and OHCs and, consequently, NIHL, as we demonstrate in adult CBA/J mice. In line with this concept, cleavage of α-fodrin (but not total α-fodrin) increases in OHCs and cochlear homogenates, supporting the activation of calpain with calcium overload, consistent with previous reports (Wang et al., 2007; Yamaguchi et al., 2017). The BBB- and cell-membrane-permeable cysteine protease inhibitor MDL-28170 is an interesting compound in this context as it not only inhibits the calcium-mediated activation of calpain but also has antioxidant and anti-inflammatory properties, desirable in attenuating noise-induced damage to cochlear sensory hair cells (Ikeda and Morizono, 1988; Fridberger et al., 1998; Ohlemiller et al., 1999; Yamashita et al., 2004; Fujioka et al., 2006; Chen et al., 2012; Hill et al., 2016; Dhukhwa et al., 2019; Fettiplace and Nam, 2019). Second, on a cellular level, MDL-28170 prevents reduction of PI3K/Akt in OHCs by exposure to noise and upregulates the PI3K/Akt cell survival signaling pathway, thus affecting multiple pathways opposing NIHL by enhancing cell survival and blocking cell death.

Attenuation of noise-induced losses of IHC ribbon synapses and OHCs and NIHL in adult CBA/J mice by MDL-28170 is likely mediated by inhibition of noise-induced apoptotic cell death. Excessive activation of calpains can indeed be responsible for apoptotic and necrotic cell death in neuronal cells (Cheng et al., 2018). For example, calpains have been shown to activate caspase-12 and caspase-3, leading to apoptotic cell death (Nakagawa and Yuan, 2000; McCollum et al., 2002). As α-fodrin is primarily cleaved by activation of caspases (Bennett and Gilligan, 1993), our data of increased α-fodrin cleavage after noise exposure in OHCs and cochlear homogenates support the notion of noise induced-apoptotic hair cell death, in agreement with our previous report (Zheng et al., 2014). Additionally, α-fodrin is a member of the spectrin family and is widely expressed as a cytoskeletal protein (Siman et al., 1984; Dutta et al., 2002). Increases in cleaved α-fodrin might also be involved in modification of cytoskeletal proteins as we have previously reported (Chen et al., 2012; Han et al., 2015). However, total α-fodrin expression does not change with noise insults, suggesting that noise exposure does not alter protein synthesis of α-fodrin.

Aside from the important role of Ca^2+^ overload in sensory hair cells and endolymph in noise-induced hair cell loss, accumulation of reactive oxygen species (ROS) and increased cytokines are well-accepted as contributors to the pathogenesis of noise-induced loss of sensory hair cells (Sha and Schacht, 2017; Wang and Puel, 2018). Interestingly, MDL-28170 has anti-inflammatory properties (Hu et al., 2019) that might add to its efficacy. In this study, DMSO as a solvent for MDL-28170 also provides some attenuation of noise-induced auditory thresholds, likely by its ability to reduce lipid peroxidation and hydroxyl radicals (Sanmartín-Suárez et al., 2011). The protective effects of DMSO are minimal but agree with studies showing that antioxidant treatment attenuates NIHL (Sha and Schacht, 2017; Wang and Puel, 2018). The use of DMSO as a solvent for MDL-28170 likely provides additive antioxidant and anti-inflammatory properties.

Finally, calpain-1 and calpain-2 reduce PI3K enzymatic activity of both the catalytic p110 and regulatory p85 subunits (Beltran et al., 2011), lowering the capacity of this cell survival pathway. We have previously shown that p85α is decreased in sensory hair cells after traumatic noise exposure and that this effect is associated with noise-induced OHC death (Chen et al., 2015). Expanding upon our previous results, p85α decreases in OHCs when assessed 3 h after noise exposure, whereas treatment with MDL-28170 not only completely blocks this reduction but increases p85α by twofold compared to control mice without exposure. Furthermore, treatment with MDL-28170 alone upregulates p85α levels even further, more than threefold in OHCs compared to controls without the exposure, the first documentation–to our knowledge–that MDL-28170 treatment robustly increases p85α in OHCs. In agreement with the known action of p85α on p-Akt (S473) and our previous data (Chen et al., 2015), noise exposure decreases p-Akt (S473) in OHCs. Treatment with MDL-28170 not only completely prevents reduction of p-Akt (S473) by noise exposure but increases p-Akt (S473) levels by over twofold in OHCs. These results suggest yet another protective action by MDL-28170, the prevention of reduced enzymatic activity of PI3K by noise-activated calpain-1 and calpain-2. Of note, we have not observed changes in p-85α and p-Akt (S473) levels by Western blot when assessed using whole cochlear homogenates 3 h after completion of exposure. This may be due to the dilution by other cell types in the homogenates.

In summary, MDL-28170 treatment prevents noise-induced hair cell loss and hearing loss by attenuating several cell-death pathways involved in the pathogenesis of NIHL and upregulating PI3K/Akt survival pathways in sensory hair cells. The multifaceted function of this compound provides a promising strategy for the prevention of NIHL.

## Data availability statement

The original contributions presented in this study are included in this article/supplementary material, further inquiries can be directed to the corresponding author.

## Ethics statement

The animal studies were reviewed and approved by the Institutional Animal Care and Use Committee of Medical University of South Carolina.

## Author contributions

All authors listed have made a substantial, direct, and intellectual contribution to the work, and approved it for publication.

## Author disclaimer

The data contained in the manuscript have not been previously published, have not been submitted elsewhere, and will not be submitted elsewhere while under review. All research protocols were approved by the Institutional Animal Care and Use Committee at the Medical University of South Carolina. Animal care was under the supervision of Division of Laboratory Animal Resources at MUSC. All authors have reviewed the contents of the manuscript, approve of its contents, and validate the accuracy of the data.
